# Metabotropic glutamate receptor-1 regulates inflammation in triple negative breast cancer

**DOI:** 10.1038/s41598-018-34502-8

**Published:** 2018-10-30

**Authors:** Rachel E. Sexton, Ali H. Hachem, Ali A. Assi, Miriam A. Bukhsh, David H. Gorski, Cecilia L. Speyer

**Affiliations:** 1 Cancer Biology Graduate Program, Wayne State University, Barbara Ann Karmanos Cancer Institute, 4100 John R St., Detroit, MI 48201 USA; 2 Central Michigan University College of Medicine, 1000 Houghton Ave., Saginaw, MI 48602 USA; 3 University of Michigan School of Medicine, M4101 Medical Science Building I — C Wing, 1301 Catherine St., Ann Arbor, MI 48109-5624 USA; 4 Oakland University William Beaumont School of Medicine, 2200 N. Squirrel Road, Rochester, MI 48309 USA; 5 Michael and Marian Ilitch Department of Surgery, Wayne State University School of Medicine, Barbara Ann Karmanos Cancer Institute, 4100 John R St., Detroit, MI 48201 USA; 6 Molecular Therapeutics Program, Barbara Ann Karmanos Cancer Institute, 4100 John R St., Detroit, MI 48201 USA; 7 Tumor Microenvironment Program, Barbara Ann Karmanos Cancer Institute, 4100 John R St., Detroit, MI 48201 USA

## Abstract

Breast cancer remains a major cause of death among women. 15% of these cancers are triple negative breast cancer (TNBC), an aggressive subtype of breast cancer for which no current effective targeted therapy exists. We have previously demonstrated a role for mGluR1 in mediating tumor cell growth, endothelial cell proliferation, and tumor-induced angiogenesis in TNBC. In this study, we explore a role for mGluR1 in regulating inflammation in TNBC. *GRM1* expression was silenced in MDA-MB-231 cells to study changes in expression of inflammatory genes regulated by mGluR1. Results were confirmed by ELISA using *GRM1*-silenced and overexpressed cells and mGluR1 inhibitors. A functional role for these differentially expressed genes was determined *in vitro* and *in vivo*. 131 genes were differentially expressed in *GRM1*-silenced MDA-MB-231 cells, with some of these falling into four major canonical pathways associated with acute inflammation, specifically leukocyte migration/chemotaxis. Upregulation of three of these genes (*CXCL1, IL6, IL8*) and their corresponding protein was confirmed by qPCR analysis and ELISA in *GRM1*-manipulated TNBC cells. Upregulation of these cytokines enhanced endothelial adhesion and transmigration of neutrophils in co-culture assays and in 4T1 mouse tumors. Our results suggest mGluR1 may serve as a novel endogenous regulator of inflammation in TNBC.

## Introduction

Approximately 15% of all breast cancer cases in the U.S. are triple negative breast cancer (TNBC), an aggressive subtype that lacks receptors for estrogen, progesterone and human epidermal growth factor receptor 2^1^. The only current systemic treatment options for TNBC include cytotoxic chemotherapeutics that target rapidly replicating cells and produce significant morbidity. The identification of new molecular targets to treat TNBC thus has the potential to produce new effective therapies for TNBC while reducing toxicity associated with standard chemotherapy and addressing a critical unmet need in breast cancer therapy.

Recently, a link between the tumor immune microenvironment (TIM) and TNBC has been established, in which increased immune infiltrates positively correlated with improved pathologic complete response, decreased distance recurrence, and improved progression-free survival^2–7^. Determining how to stimulate the influx of these immune infiltrates could potentially reduce mortality associated with TNBC. However, the immune system can play a dual role in cancer, acting both as a suppressor or promoter of tumor growth. The system is complex and involves various factors secreted by tumor cells, surrounding stromal and invading immune cells^8^. In breast cancer, immune cells, including tumor-associated neutrophils (TANs) play a major role in determining tumor cell fate through expression of various inflammatory agents, including chemokines^9–14^. Thus, polarizing the TIM in favor of an anti-tumor phenotype could potentially be effective as a treatment for TNBC.

Previously, we identified metabotropic glutamate receptor-1 (mGluR1) as a possible therapeutic target in TNBC. Metabotropic glutamate receptors (mGluRs) are a family of G-protein coupled receptors known to mediate reflexes in the nervous system^15^. mGluR1 belongs to the Group I mGluR family whose over-expression has been linked to melanoma^16,17^. We detected high levels of mGluR1 in various TNBC cells compared to normal breast epithelial cells and observed inhibiting or silencing mGluR1 inhibits breast cancer growth and angiogenesis, both *in vitro* and *in vivo*^18–20^. In addition, we have demonstrated *GRM1* and mGluR1 are expressed at significantly higher levels in human breast cancer tissue compared to patient-derived normal breast tissue^18^. In this study, we observe mGluR1 as an inhibitor of both the production of inflammatory chemoattractants by TNBC cells as well as the induction of neutrophil (PMN) transmigration. These findings suggest mGluR1 may serve as a novel endogenous regulator of inflammation in TNBC by initiating signals in breast cancer cells that modulate PMN transmigration and function within the TIM.

## Results

### GRM1 mediates inflammatory signaling pathways

**Figure 1:**
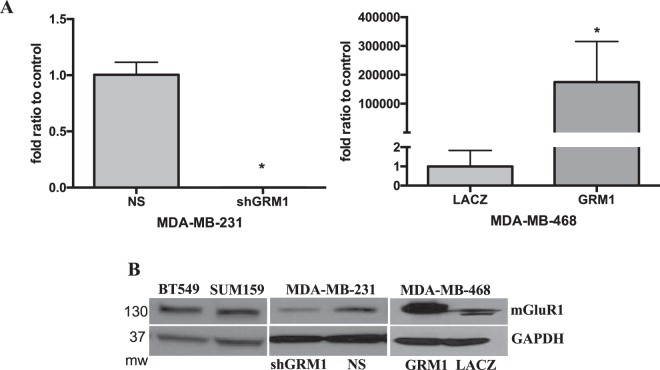
*GRM1* and mGluR1 expression in TNBC. (**A**) Knockdown of *GRM1* was accomplished by infecting MDA-MB-231 cells with GIPZ shRNA Lentiviral vectors containing a puromycin resistance gene and shRNA against *GRM1* or a non-silencing shRNA construct (NS). *GRM1* overexpression was accomplished by infecting MDA-MB-468 cells with pLenti6.3/V5-TOPO vectors containing a blasticidin resistance gene and *GRM1* or *LACZ* construct (*LACZ*). Cells were stably selected with puromycin (1 μg/ml) or blasticidin (5 μg/ml) for 7 days and levels of *GRM1* message (**A**) or its corresponding protein, mGluR1 (**B**) were measured by QPCR or Western blot, respectively. mGluR1 expression in SUM159, BT549 and non-transduced MDA-MB-231 TNBC cell lines were also detected. Results in **A** represent n = 2 experiments and are expressed as the mean ± SEM where *is P < 0.05 compared to NS cells.

**Figure 2:**
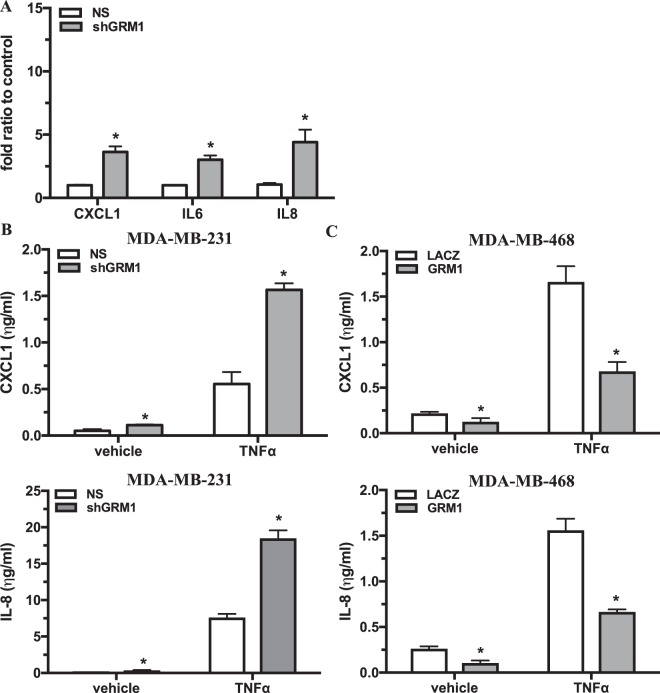
*GRM1* mediates CXCL1, IL-6 and IL-8 expression in TNBC cells. Knockdown of *GRM1* in MDA-MB-231 cells induced a significant increase in *CXCL1*, *IL6*, and *IL8* gene expression determined by QPCR (**A**) as well as the corresponding proteins CXCL1 and IL-8 determined by ELISA either alone or in the presence of TNFα (10 ng/ml) for 24 hours (**B**,**C**) *GRM1* overexpression in MDA-MB-468 cells induce a significant decrease in CXCL1 and IL-8 proteins levels determined by ELISA, either alone or in the presence of TNFα (10 ng/ml) for 24 hours. All results are expressed as the mean ± SEM of n = 3 experiments performed in triplicate where *is P < 0.05 compared to their respective vehicle control cells.

Microarray gene expression analysis was performed using *GRM1*-silenced MDA-MB-231cells (Fig. 1). After stable selection and plating of cells for 24 hours, 131 genes were differentially expressed in the *GRM1*-silenced cells (see Supplementary Data S1) compared to NS cells^21^. A grouping of these genes fall into four major canonical pathways associated with acute inflammation (Table 1). Further analysis of these genes using the DAVID tool show they map to categories associated with leukocyte migration/chemotaxis (Table 2). qPCR analysis confirmed expression of three of these genes, *CXCL1, IL6*, and *IL8* which were significantly upregulated in the *GRM1*-silenced compared to NS cells by >3-fold for *CXCL1* and *IL6* and >4-fold for *IL8* (Fig. 2A). Protein levels for both CXCL1 and IL-8 were also measured by ELISA and shown to be low but significantly upregulated after *GRM1* silencing (Fig. 2B). Since protein levels for both of these chemokines were expressed at low levels, the cells were also treated for 24 hours with TNFα, a cytokine known to be present in the TIM^22^. Treatment with TNFα alone induced a dramatic increase in both CXCL1 and IL-8 secretion that was significantly increased by over 2-fold and 3-fold, respectively, in the *GRM1*-silenced cells compared to NS cells.

**Table 1:** Canonical pathways and genes regulated by *GRM1* in MDA-MB-231 cells.

Canonical Pathway	P-value	Observed genes
Cytokine receptor degradation signaling	3.92E-03	PRKCA, IL8, MAP3K8, IRAK1, PAK2, CSF2, IFNAR1, IL6
IL23-mediated signaling events	5.43E-03	CCL2, CXCL1, IL6
Ras signaling in the CD4+ TCR pathway	8.01E-03	PRKCA, MAP3K8
Raf activation signaling (through RasGRP)	8.84E-03	HLA-DQB1, LOC100133583, HLA-DRB4

**Table 2:** Gene Ontology Biological Process terms over-represented in *GRM1* silenced MDA-MB-231 cells compared to NS cells.

Biological Process Term	Bonferroni P-value
leukocyte migration	4.77E-06
sterol biosynthetic process	1.57E-05
leukocyte chemotaxis	1.97E-05
cell chemotaxis	2.44E-05
isoprenoid metabolic process	3.96E-05
neutrophil chemotaxis	6.24E-05
acute inflammatory response	6.75E-05
chemotaxis	6.82E-05
sterol metabolic process	7.79E-05
isoprenoid biosynthetic process	8.67E-05
positive regulation of response to external stimulus	1.74E-04
cholesterol biosynthetic process	1.94E-04
regulation of inflammatory response	3.38E-04
lymphocyte chemotaxis	3.92E-04
~regulation of defense response	3.97E-04
steroid biosynthetic process	5.18E-04
carboxylic acid biosynthetic process	5.74E-04
macrophage chemotaxis	6.69E-04
blood coagulation	0.001028087
cell migration	0.001269035
steroid metabolic process	0.001876937
GO:0019752 ~ carboxylic acid metabolic process	0.002759414
GO:0007584 ~ response to nutrient	0.003280091
GO:0051607 ~ defense response to virus	0.003421269
GO:0031349 ~ positive regulation of defense response	0.00401224
GO:0050921 ~ positive regulation of chemotaxis	0.007128379
GO:0050729 ~ positive regulation of inflammatory response	0.007616178
GO:0050920 ~ regulation of chemotaxis	0.008118657
GO:0030168 ~ platelet activation	0.008635684
GO:0048520 ~ positive regulation of behavior	0.00971285

To further confirm a role for *GRM1* in mediating CXCL1 and IL-8 production in TNBC cells, low *GRM1* expressin*g* MDA-MB-468 cells were transduced to overexpress *GRM1* or its corresponding *LACZ* control vector (Fig. 1A,B) and protein levels for both CXCL1 and IL-8 were measured by ELISA after stable selection with blasticidin. Both CXCL1 and IL-8 protein levels were significantly down-regulated in the *GRM1* overexpressing cells compared to *LACZ* cells (Fig. 2C). Treatment with TNFα induced a significant increase in both CXCL1 and IL-8 secretion that was significantly inhibited by greater than 60% in the *GRM1* overexpressed cells compared to *LACZ* cells (Fig. 2C). Since the role of IL-6 in mediating PMN adhesion/migration is controversial, with recent findings suggesting it is not a direct regulator of PMN function^23^, IL-6 protein levels were not examined.

**Figure 3:**
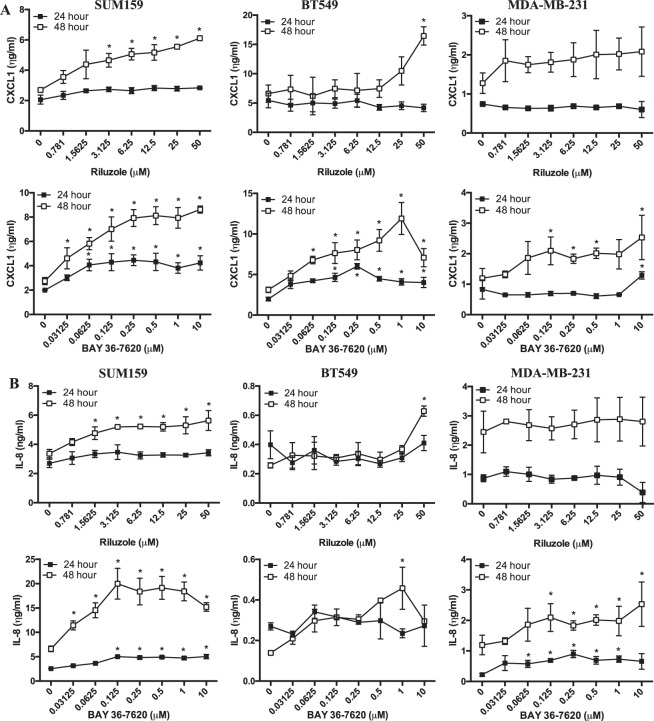
Riluzole and BAY36-7620 increase cytokine protein expression in TNBC cells. Effect of riluzole and BAY36-7620 on CXCL1 (**A**) and IL-8 (**B**) secretion from SUM159, BT549 and MDA-MB-231 cells treated for 24 or 48 hours. Results represent the mean ± SEM of n = 2 experiments, performed in triplicate, where *is P < 0.05 compared to vehicle treated cells.

mGluR1-mediated regulation of CXCL1 and IL-8 was further demonstrated using mGluR1 inhibitors BAY36-7620 (BAY) and riluzole in BT549, SUM159 and MDA-MB-231 cells, which also express mGluR1 (Fig. 1B). All 3 cell lines secreted high levels of CXCL1 by 24 hours but did not increase dramatically between 24 and 48 hours (Fig. 3A). After 24 hours, riluzole had no significant effect on CXCL1 levels in any cell line. This is consistent with microarray analysis performed previously with riluzole-treated MDA-MB-231 cells^24^. By 48 hours, a dose-dependent increase in CXCL1 was observed in all 3 cell lines with a significant increase of over 3-fold in SUM159 and BT549 cells after treatment with the highest dose (50 μM). The effect of riluzole on MDA-MB-231 CXCL1 levels was not significant. Unlike riluzole, after treatment with BAY, a dose-dependent increase in CXCL1 levels did occur in both SUM159 and BT549 cells by 24 hours with a significant increase of 2-fold at the highest dose tested (10 μM). MDA-MB-231 cells were not as responsive, with a small but significant increase only at the highest dose. However, at 48 hours, these levels continued to increase with BAY significantly increasing CXCL1 levels by up to 3-fold in all 3 cell lines.

Unlike CXCL1, IL-8 chemokine secretion levels varied between cell lines with SUM159 expressing high levels at 24 hours with less detection in the BT549 (just above background) and MDA-MB-231 cells (Fig. 3B). After 24-hour treatment with riluzole, no significant increase in IL-8 secretion was detected in any cell line, consistent with previous microarray results in riluzole treated MDA-MB-231 cells^24^. However, after 48 hours, a dose-dependent increase in IL-8 levels were observed in SUM159 cells with a significant increase of about 1.5-fold at the highest dose tested (50 μM). BT549 cells were unresponsive to lower levels of riluzole but did induce a significant 2.5-fold increase at the highest dose tested. MDA-MB-231 cells were still unresponsive to riluzole. The response of SUM159 and MDA-MB-231 cells to BAY was much more robust with a dose-dependent increase in IL-8 levels as early as 24 hours and still evident after 48 hours. At the highest dose tested (10 μM), BAY induced a significant increase of at least 2-fold at both times points. Similar to riluzole treatment, BT549 cells were unresponsive to lower levels of BAY but a significant increase of over 2-fold was observed at higher levels (0.5–1.0 μM).

### In vitro and in vivo regulation of neutrophil chemotaxis

**Figure 4:**
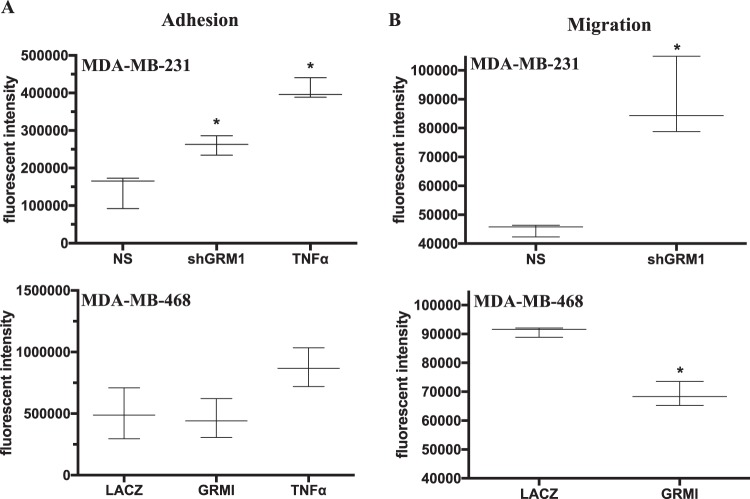
Effects of *GRM1* expression on PMN adhesion and migration *in vitro*. (**A**) HMEC-1 cells were grown to confluence in 96-well black culture plates and exposed to 24 hour conditioned medium from NS and *GRM1*-silenced MDA-MB-231 cells or *LACZ* and *GRM1*-overexpressed MDA-MB-468 cells for 30 minutes or to TNFα (10 ng/ml) for 6 hours as a positive control. The medium was removed and BCECF-AM labeled PMNs (see Methods section) were added at 2 × 10^5^/well and allowed to attach for 30 minutes. Unattached PMNs were removed by washing in PBS and adhered PMNs detected by FITC fluorescence. (**B**) HMEC-1 cells were grown to confluence on BD cell culture inserts (8 μm pore size) in 24-well plates. Medium in bottom of wells were replaced with 24 hour conditioned medium as described above and labeled PMNs were added (2 × 10^6^ per insert) and allowed to migrate. After 1.5 hours, medium was removed and PMNs isolated by centrifugation and detected by measuring FITC fluorescence. For both (**A**,**B**), results are the mean ± SEM of n = 3 experiments, performed in triplicate where *is P < 0.05 compared to endothelial monolayers treated with their respective NS or *LACZ* conditioned medium.

CXCL1 and IL-8 regulate PMN adhesion and transmigration during inflammation^25,26^. This process is triggered by binding of chemokine to its receptor on PMNs, causing integrin clustering on the cell surface of the PMN, resulting in increased binding of integrin to ICAM-1 on the endothelium. Following these events, PMNs migrate across the endothelium. mGluR1’s ability to regulate this process was examined. Endothelial monolayers were exposed for 30 minutes to conditioned medium from either *GRM1*-silenced or overexpressed cells (cultured for 24-hours) prior to adding labeled PMNs for 30 minutes. This incubation process allows for chemokines expressed in the medium to bind their receptors on the endothelium^26–30^. After 30 minutes, monolayers were washed to remove non-adherent PMNs and remaining PMNs were detected by fluorescence. As a positive control, monolayers were treated with TNFα for 6 hours. As expected, treatment of the endothelium with TNFα induced an almost 2-fold and 3-fold increase in PMNs adhering to the monolayers compared to NS or LACZ controls (Fig. 4A). Treatment of monolayers with medium from *GRM1* silenced MDA-MB-231 cells significantly increased PMN binding by almost 2-fold compared to treatment with NS medium. In contrast, treatment of monolayers with medium from *GRM1* overexpressed MDA-MB-468 cells decreased binding of PMN but this effect was small and insignificant compared to treatment with *LACZ* control medium.

Following adhesion to the vascular endothelium, PMNs will transmigrate across the monolayer moving towards a chemokine gradient. To examine a role for mGluR1, inserts containing endothelial monolayers were placed in medium from either *GRM1* silenced or over-expressed cells and labeled-PMNs were placed on top of the endothelial monolayer and allowed to transmigrate for 90 minutes. Similar to the adhesion assay, there was a significant (almost 2-fold) increase in PMNs present in the medium from *GRM1* silenced cells compared to NS medium (Fig. 4B). In the medium from *GRM1* overexpressed cells, there was a small (23%) but significant decrease in the number of PMNs present compared to medium from *LACZ* cells.

**Figure 5:**
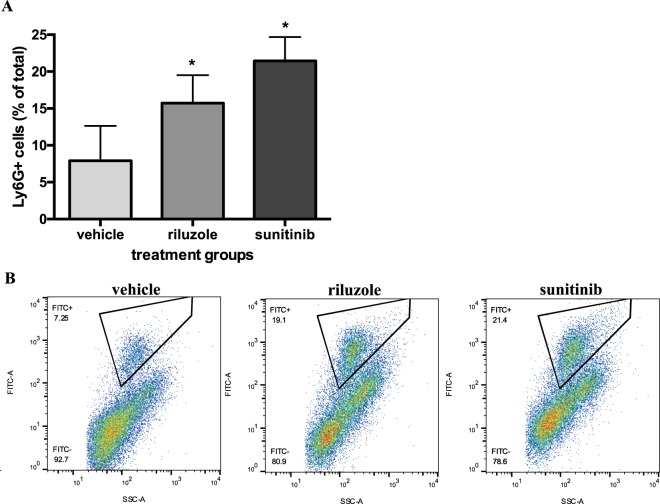
Riluzole inhibits migration of PMN into tumors in a 4T1 mouse model. 4T1 mouse TNBC cells were injected into the mammary fat pads of BALB/c mice and allowed to grow until tumors reached a mean size of 100 mm^3^ and then divided into groups of 6 and treated daily with either vehicle (DMSO), riluzole (18 mg/kg) or sunitinib (20 mg/kg) as a positive control, for 14 days. Tumors were measured and digested in collagenase/DNase and TANs detected by FACS analysis labeling with anti-Ly6G FITC-conjugated antibody. Both riluzole and sunitinib significantly increased tumor PMN presence (**A**) which coincided with tumor burden as previously published^19^. Results are the mean ± SEM where *is P < 0.05 compared to vehicle treated mice. (**B**) Representative scatter plot of treated cells staining positive for Ly6G^+^ (FITC^+^).

To test whether mGluR1 can regulate PMN migration into tumors, we used the *GRM1*-expressing 4T1 mouse tumor model to measure PMN presence in the tumors after injections with riluzole or sunitinib, an anti-angiogenic drug known to regulate PMN migration^31,32^. As our previously published growth curve demonstrates^19^, treatment with riluzole or sunitinib significantly decreased tumors by 50% compared to vehicle DMSO. In this same study, we measured PMN presence and found decreased tumor volume corresponded with a significant increase in PMN presence, detected by anti-Ly6G positive staining (Fig. 5A,B). In riluzole-treated mice, there was a significant 2-fold increase in Ly6G positive staining PMNs in the tumors compared to vehicle treated tumors.

## Discussion

In this study, we implicate mGluR1 as a novel endogenous inhibitor of both the production of inflammatory chemoattractants by TNBC cells as well as the induction of neutrophil (PMN) transmigration. This was demonstrated in various TNBC cells where inhibition of mGluR1 using either the mGluR1 inhibitors (BAY36-7620, riluzole) or an shRNA directed at mGluR1 increased expression of CXCL1 and IL-8 and overexpressing *GRM1* had strong inhibitory effects. Since CXCL1 and IL-8 are strong PMN chemoattractants^26,33^, a role for mGluR1 in regulating PMN transmigration was examined both *in vitro* and *in vivo*. *In vitro*, exposure of endothelial cells to conditioned medium from sh*GRM1* MDA-MB-231 cells resulted in increased migration of PMNs through endothelial monolayers whereas conditioned medium from *GRM1* overexpressing MDA-MB-468 cells had a strong inhibitory effect. Interestingly, even though CXCL1 and IL-8 secretion levels were significantly decreased in *GRM1* overexpressing MDA-MB-468 cells, PMN adhesion was not significantly affected. This suggests high ectopic expression of mGluR1 may trigger release of other molecules such as TNF, IFN or TGFβ from the TNBC cells or the endothelial cells themselves^22,34,35^ that are known to inhibit PMN transmigration.

*In vivo*, we also observed that treating the syngeneic mouse 4T1 mammary cancer model with riluzole increased the presence of PMNs in the tumors, which coincided with decreased tumor growth^19^. This suggests that mGluR1, in addition to its direct effect on tumor cell growth and survival^18–20^ is capable of regulating inflammation within the TIM. However, recent findings suggest riluzole’s anti-tumor properties in breast cancer are likely largely mediated through mGluR1 independent mechanisms^36^. In that study, riluzole inhibited cell growth, invasion and migration in both *GRM1* silenced and over-expressed cells suggesting lack of *GRM1* involvement. In addition, a recent microarray analysis demonstrated cell cycle genes to be major pathways regulated by riluzole further suggesting alternative pathway(s) by which riluzole functions other than mGluR^24^. Further *in vivo* studies utilizing more specific mGluR1 inhibitors, such as BAY36-7620, are necessary to definitively define a role for mGluR1 in mediating inflammation within the TIM. Nonetheless, riluzole’s ability to mediate PMN migration in the 4T1 tumor model in the present study is important because riluzole is an already FDA-approved drug that can be quickly translated into the clinic.

The role of inflammation in cancer is complex, demonstrating both pro- and anti-tumor properties. Although acute inflammation is associated with anti-tumor immune responses^37,38^ a link between chronic inflammation and neoplastic progression has long been recognized^39–41^. Within the TIM, there is a complex mix of cell types contributing inflammatory factors including cancer cells themselves. TANs were originally thought to promote cancer by affecting angiogenesis^11,42^ or by modulating the TIM in favor of immunosuppression. However, based on recent evidence^43–45^, it appears the TIM can be manipulated to polarize TANs to acquire anti-tumor phenotypes involving CXCL1 and TNFα^22,31,32,46–48^. TNBC cell’s ability to regulate PMN adhesion and migration through production of these chemoattractants strongly confirms these studies and suggests mGluR1 as a novel endogenous regulator of leukocyte phenotype within the TIM.

In addition to PMNs, data from the microarray analysis show lymphocyte migration and chemotaxis to be strongly upregulated by *GRM1* silencing (95-fold increase). Recent studies demonstrate a strong association of TIL presence with increased metastasis-free survival and decreased distant recurrence in early stage TNBC^49,2,5^. These studies correlate with other studies involving TANs where in early-stage tumors, TANs possess anti-tumorigenic properties and actually activate TILs^50–52^. Thus, it appears targeting mGluR1 early in the treatment regime may play an important role in stimulating an adaptive immune response in TNBC. In support of this, riluzole has been shown to increase survival of CD8 T-cells in HIV-1-infected individuals and enhance proliferation of anti-CD3/CD28 stimulated T cells^53^.

Previously, we identified mGluR1 as a promising target for breast cancer therapy based on its roles promoting angiogenesis and tumor cell growth. Our results now implicate mGluR1 and riluzole as novel endogenous inhibitors of inflammation and PMN transmigration in TNBC. Further studies into the mechanism by which mGluR1 and riluzole mediate these effects could be very useful in the development of therapeutic targets for treating TNBC cancer and would provide more insight into the role inflammation plays on the progression of TNBC.

## Materials and Methods

### Reagents and Cell Culture

Cell culture reagents were purchased from Thermofisher Scientific (Waltham, MA). TNBC cell lines were purchased from ATCC except the SUM159 cell line was a kind gift from Stephen Ethier (Medical University of South Carolina). The mouse 4T1-12B cell line was a kind gift from Fred Miller^54^. The human microvascular endothelial cell line HMEC-1 was obtained from Centers for Disease Control and cultured as described previously^19^. Cell lines were authenticated via cytogenetic analysis or used within 6 months of purchase or stored in liquid nitrogen. The specific mGluR1 inhibitor, BAY36-7260, and riluzole were purchased from Tocris Bioscience (Minneapolis, MN). All tumor digestion reagents were purchased from Sigma Aldrich (St. Louis, MO).

### Stable transduction of cells with GRM1 shRNA or GRM1 plasmids

Reagents for transduction assays were purchased from ThermoFisher Scientific. GIPZ Lentiviral particles containing *GRM1* shRNA vector (sh*GRM1*) or non-silenced control vector (NS) were obtained from Karmanos Cancer Institute by subscription to Thermo Scientific GIPZ shRNAmir library. Lentiviral particles containing these vectors were generated by reverse transfection of these constructs with Trans-Lentiviral package mix, into HEK293T cells using Arrest-In/Express-In transfection reagent. Approximately 10^6^ TU/ml was used to infect MDA-MB-231 cells in the presence of polybrene (10 μg/ml) and stable cultures generated by growing in puromycin (1 μg/ml) as previously described^18–20,36^. *GRM1* silencing was confirmed by Western blot and RT-QPCR.

Construction of Lentiviral *GRM1* vectors has been described previously^18–20,36^. Lentiviral particles containing the *GRM1* vector or LACZ control vector were generated by reverse transfection of these constructs with Virapower packaging mix into HEK293T cells using Lipofectamine 2000 reagent. A dilution (1:1) of viral supernatant was used to infect low *GRM1*-expressing MDA-MB-468 cells using polybrene (10 μg/ml). Stable cultures were generated by growing in blasticidin (5 μg/ml).

### Microarray analysis of mGluR1 mediated pathways

After stably selecting *GRM1* silenced or NS MDA-MB-231 cells using puromycin (10 μg/ml) for two weeks, cells were plated in triplicate overnight and RNA extracted using RNeasy Plus Mini Kit (Qiagen, Valencia, CA). RNA was quality assessed using the 2100 Bioanalyzer System and hybridized to the Illumina® Human HT-12v4 array. Data was uploaded to BeadStudio, background-corrected and normalized using rank invariant algorithm. Differentially expressed genes were identified using Illumina Custom Error Model and genes differentially expressed were uploaded to Genomatix software suite to determine over-represented canonical pathways. The online DAVID tool was used to determine Gene Ontology Biological Process terms over-represented by the data.

### RT-QPCR analysis

Total RNA was extracted using Qiagen’s RNeasy Plus Mini Kit and reverse transcription performed using High Capacity cDNA Reverse Transcription Kit (Thermo Scientific) according to manufacturer protocols. QPCR was performed using ABsolute QPCR Mix with ROX (Thermo Scientific) according to manufacturer using the sense/anti-sense primers for *GRM1, CXCL1, IL6, IL8*, and housekeeping gene *GAPDH* as listed in Table 3 below.

**Table 3:** Sense/Anti-sense primers used in qPCR experiments.

*GRM1*	Sense:	5′-GCA CGG CCT GCA AAG AGA ATG AAT-3′
	Anti-Sense:	5′-TCC ACT CAA GAT AGC GCA CAG GAA-3′
*CXCL1*	Sense:	5′- GAA AGC TTG CCT CAA TCC G-3′
	Anti-Sense:	5′- CAC CAG TGA GCT TCC TCC C-3′
*IL6*	Sense:	5′- AGG AGA CTT GCC TGG TGA AA-3′
	Anti-Sense:	5′- AAA GCT GCG CAG AAT GAG AT-3′
*GAPDH*	Sense:	5′-ACA ACT TTG GTA TCG TGG AAG G-3′
	Anti-Sense:	5′-CAG TAG AGG CAG GCA TGA TGT TC-3′

Thermal cycling was performed as previously described^20^. Controls without RT were used to confirm lack of genomic DNA. Relative fold change in *GRM1, CXCL1, IL6*, or *IL8* expression compared to NS or *LACZ* control was determined using the following equation: 2^−ddCt^, where −ddCt is difference between the dCt of the cytokine gene and the housekeeping gene normalized to the control values.

### Protein Expression

30–60 μg of protein isolated from TNBC cells was separated by SDS-polyacrylamide gel electrophoresis (10%) and transferred to PVDF membranes. Immunodetection of mGluR1 was performed using anti-mGluR1 antibody (Alamone Labs, Jerusalum, Israel) with appropriate secondary antibody and detected by chemiluminescence. Blots were further reprobed with antibody against GAPDH (Novus Biologics, Littleton, CO).

### Quantification of Cytokine Production by ELISA

CXCL1 and IL-8 levels in culture supernatants from stably transduced cells or after treatment with TNFα (10 ng/ml), BAY36-7620, riluzole, or vehicle (0.01% DMSO) were measured by sandwich ELISA (R&D systems, Minneapolis, MN) according to manufacturer protocol. Since these inhibitors are known to inhibit cell growth, relative TNBC cells numbers were determined after each experiment using MTT analysis^20,36^, and chemokine expression was normalized to cell counts.

### Syngeneic breast tumor model

4T1 cells (3 × 10^4^) were injected into mammary fat pads of female BALB/c mice (Harlan Laboratories, age 6–8 weeks) and allowed to grow until tumors reached a mean volume of 62 mm^3^ (approximately 10 days). Mice were then divided into groups of 10 and treated daily with i.p. injections of riluzole (18 mg/kg), sunitinib (20 mg/kg), or vehicle (DMSO) for 14 days. Tumor size was measured three times a week using a Vernier caliper and tumor volume estimated using the following formula: length × width × depth/2. After 14 days of treatment, mice were euthanized and their tumors harvested, minced, and digested in enzyme solution (1 g tissue/10 ml solution) containing collagenase type IV (0.15 mg/ml), collagenase type I (0.4 mg/ml), DNase I (1.25 mg/ml) and BSA (0.5%) for 1 hour. PMNs were identified as described below. Animals were housed in a pathogen-free facility and all animal studies were performed in accordance with local IACUC at Wayne State University.

### Isolation and Detection of PMN from Whole Blood and Tumors

Heparin anti-coagulated blood was obtained from healthy volunteers after informed consent and in accordance with ethical guidelines of Wayne State University. PMNs were isolated using Ficoll-Paque followed by dextran (1%) density gradient centrifugation as described previously^55^ and fluorescein-labeled with BCECF-AM (Molecular Probes, Eugene, OR) for 30 minutes at 37 °C. The percentage of PMNs in the tumor digestion mixture was determined by FACS analysis after washing mixture through a 70 μm nylon strainer and labeling with FITC-conjugated anti-Ly6G antibody (BioLegend, San Diego, CA).

### Adhesion Assays

HMEC-1 cells were grown to confluence in 96-well black plates and exposed for 30 minutes to conditioned medium from stably transduced cells (cultured for 24 hours) or to TNFα (10 ng/ml) for 6 hours as a positive control. After incubation, the medium was removed and labeled PMNs added to HMEC-1 monolayers at 2 × 10^5^/well and allowed to attach for 30 minutes. After 30 minutes, HMEC-1 monolayers were washed 3 times with PBS and remaining PMNs detected by measuring FITC fluorescence using BioTek Synergy 2 plate reader.

### Transmigration Assays

HMEC-1 cells were grown to confluence on BD cell culture inserts (8 μm pore size) in 24-well plates containing EGM-2 complete medium. Upon confluence, medium in bottom of wells was replaced with 24 hour conditioned medium from stably transduced cells, after which labeled PMNs were added (2 × 10^6^ per insert) and allowed to migrate for 1.5 hours. Media in the bottom of wells was then removed and PMNs collected by centrifugation, resuspended in PBS and detected by FITC fluorescence.

### Statistical Analyses

Differentially expressed genes were identified using the Illumina Custom Error Model where n = 3 repeats. A p-value was associated with every differential call and genes with p-value > 0.05 were discarded. In addition, genes were discarded if fold-change in expression was <1.3. Numerical data was analyzed using GraphPad Prism (v.7.0) for Macintosh. All numerical results are expressed as mean ± SEM and statistical analysis performed by one-way or two-way repeated-measures analysis of variance (ANOVA) followed by multiple comparison procedure with Student-Newman Keuls method. A value of p ≤ 0.05 was considered significant.

### Ethical approval

All applicable international, national, and/or institutional guidelines for the care and use of animals were followed. All procedures performed in studies involving animals were in accordance with the ethical standards of the institution or practice at which the studies were conducted. Animal studies were approved by the local Institutional Animal Care and Use Committee (IACUC) at Wayne State University which is structured and operated in accordance with NIH’s Office of Laboratory Animal Welfare (OLAW) *Public Health Service Policy on Humane Care and Use of Laboratory Animals* (Public Health Service NIH Assurance number D16-00198).

All procedures performed in studies involving human participants were performed with their consent and in accordance with the Declaration of Helsinki and have been approved following Expedited Review (IRB #123016MP4E) by the Chairperson for the Wayne State University Institutional Review Board (MP4).

## Electronic supplementary material

Supplementary Information File

Dataset 1

## Data Availability

The dataset supporting the conclusions of this article is available in the GEO repository, (accession # GSE106100: https//www.ncbi.nlm.nih.gov/geo/query/acc.cgi?acc=GSE106100) and is also included within the article (and in Supplementary Data S1).
