# Supplementary material for: Metabotropic glutamate receptor-1 regulates inflammation in triple negative breast cancer
Source: Sci Rep. 2018 Oct 30;8:16008. doi: 10.1038/s41598-018-34502-8 (PMC6207734; doi:10.1038/s41598-018-34502-8)
Supplement: Supplementary file 1 — Supplementary Information File [file 41598_2018_34502_MOESM1_ESM.pdf]

Title: Metabotropic glutamate receptor-1 regulates inflammation in triple negative breast cancer

Authors:
Rachel E. Sexton
Ali H. Hachem
Ali A. Assi
Miriam A. Bukhsh
David H. Gorski
Cecilia L. Speyer

SUM159 and BT549 GluR1 gel

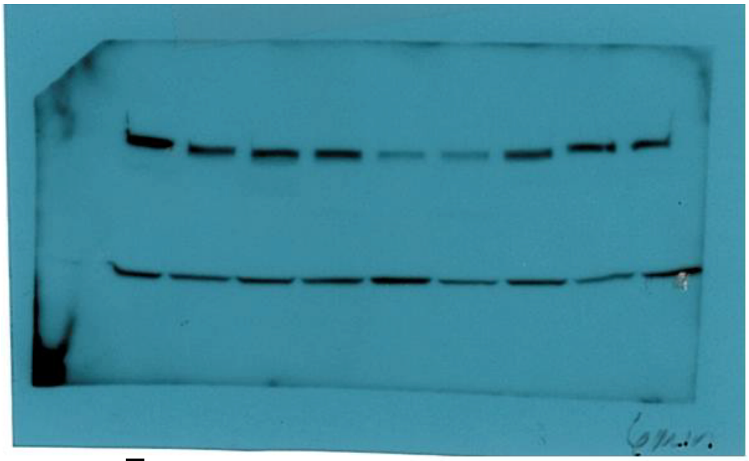

MDA-MB-231 mGluR1-silenced gel

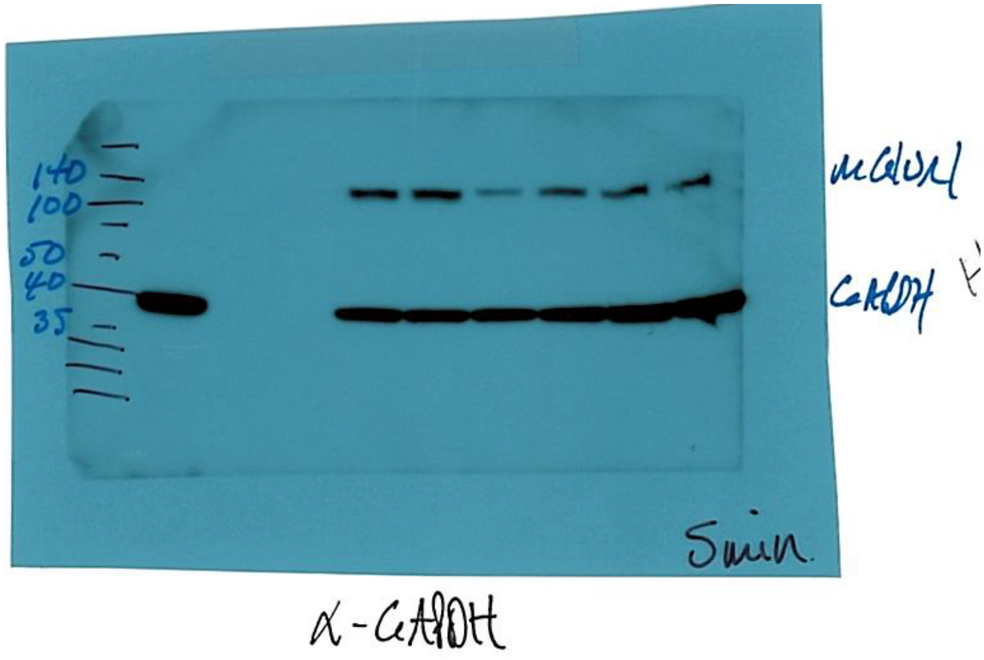

MDA-MB-468
GluR1-overexpressed cells

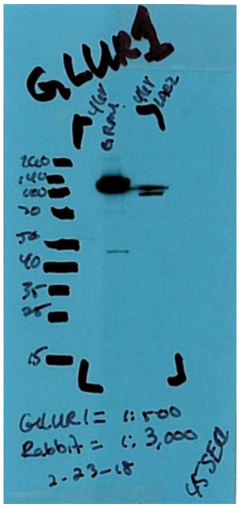

mGluR1

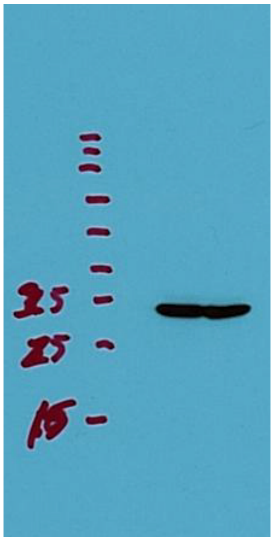

GAPDH
